# Laparoscopic left hepatectomy for a patient with intrahepatic cholangiocarcinoma metastasis in the falciform ligament: a case report

**DOI:** 10.1186/s12893-021-01115-4

**Published:** 2021-03-08

**Authors:** Yoriko Nomura, Hisamune Sakai, Jun Akiba, Toru Hisaka, Toshihiro Sato, Yuichi Goto, Masanori Akashi, Shogo Fukutomi, Daisuke Muroya, Hiroki Kanno, Shusuke Okamura, Yuta Yano, Hirohisa Yano, Yoshito Akagi, Koji Okuda

**Affiliations:** 1 Department of Surgery, Kurume University Hospital, Kurume, Japan; 2 Department of Diagnostic Pathology, Kurume University Hospital, Kurume, Fukuoka Japan; 3 Division of Gastroenterology, Department of Medicine, Kurume University School of Medicine, Kurume, Japan; 4 Department of Pathology, Kurume University School of Medicine, Kurume, Fukuoka Japan

**Keywords:** Falciform ligament, Hematogenous metastases, Intrahepatic cholangiocarcinoma, Liver, Segment IV

## Abstract

**Background:**

Intrahepatic cholangiocarcinoma (ICC) is primary cancer of the liver with poor prognosis because of its high potential for recurrence and metastasis. We experienced a rare case of ICC with hematogenous metastasis to the falciform ligament. We aimed to clarify the route of metastasis to the mesentery by increasing the accuracy of preoperative imaging and establish a hepatectomy to control cancer.

**Case presentation:**

An 85-year-old woman was referred to our hospital for a detailed study of progressively increasing liver tumors. She had no subjective symptoms. Her medical history showed hypertension, aneurysm clipping for cerebral hemorrhage, and gallstones. A detailed physical examination and laboratory data evaluation included tumor markers but did not demonstrate any abnormalities. On computed tomography scan, contrast-enhanced ultrasound, and magnetic resonance imaging with gadolinium ethoxybenzyl diethylenetriamine penta-acetic acid, the tumor appeared to be located in liver segment IV, protruding outside the liver. It appeared to contain two distinct components; we suspected ICC in the intrahepatic tumor component. Laparoscopic observation revealed that the extrahepatic lesion was an intra-falciform ligament mass; laparoscopic left hepatectomy was performed. Microscopically, the main tumor in segment IV was 15 mm in diameter and was diagnosed as moderately and poorly differentiated ICC. The tumor of the intra-falciform ligament was not continuous with the main intrahepatic nodule and was also diagnosed as ICC with extensive necrosis. There were no infiltrates in the round ligament of the liver, and several tumor thrombi were found in the small veins of the falciform ligament.

**Conclusions:**

To date, there have been a few reports of metastases of primary liver cancer to the falciform ligament. At the time of preoperative imaging and pathological diagnosis, this case was suggestive of considering that the malignant liver tumor might be suspected of metastasizing to the falciform ligament. Our case improves awareness of this pathology, which can be useful in the future when encountered by hepatic specialists and surgeons.

## Background

Intrahepatic cholangiocarcinoma (ICC) is an aggressive primary cancer of the liver and is the second most common primary liver cancer after hepatocellular carcinoma, ICC rates are increasing worldwide. Currently, surgical resection is the only curative therapy and results in 5-year overall survival rates between 15 and 40% [1]; however, up to two-thirds of patients experience recurrence after resection. This high recurrence rate causes a poor prognosis. Risk factors reported to influence survival include tumor marker, size and number, vascular invasion, lymph node metastasis; direct invasion, and local intrahepatic metastasis [2].

In this paper, we present a case in which a very small ICC had metastasized hematogenetously already to the falciform ligament of the liver. From this case, we were able to examine the pathway of translocation from the liver to the mesentery. An understanding of the pathogenesis of vascular invasion can improve preoperative diagnosis, and help establish a surgical method for controlling the hematogenous metastasis of ICC.

## Case presentation

An 85-year-old woman who had no history of chronic liver disease (viral infection and cirrhosis) was being followed-up for gallbladder polyps and was found to have a tumor in the liver. The patient was referred to our hospital because the tumor size was increasing. She had no subjective symptoms, abdominal pain, or fever. Her medical history showed hypertension, aneurysm clipping for cerebral hemorrhage, and gallstones. She had no history of smoking or drinking habit. There was no noteworthy family history.

A detailed physical examination did not demonstrate any cardiovascular or pulmonary abnormalities. Laboratory data for all parameters, including tumor markers, were normal.

**Fig. 1:**
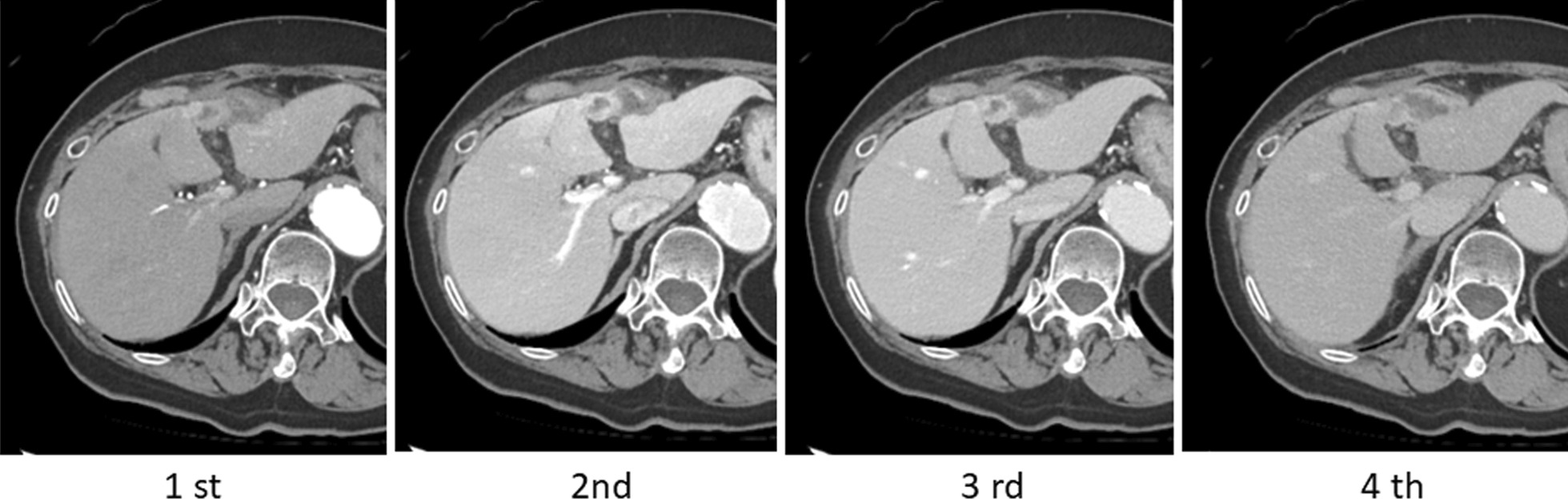
Computed tomography images of the tumor. The tumor protrudes outside the liver and appears to contain two distinct components

Computed tomography (CT) scan showed that the tumor protruded outside the liver and appeared to contain two distinct components. The tumor component in segment IV in the liver was strongly enhanced, especially in the peripheral rim in the early and delayed arterial phase, followed by progressive hyperattenuation during the late phase. However, the extrahepatic protruding area was enhanced only in the surrounding area; no enhancement was observed inside, suggesting an abscess-like cystic structure (Fig. 1).

**Fig. 2:**
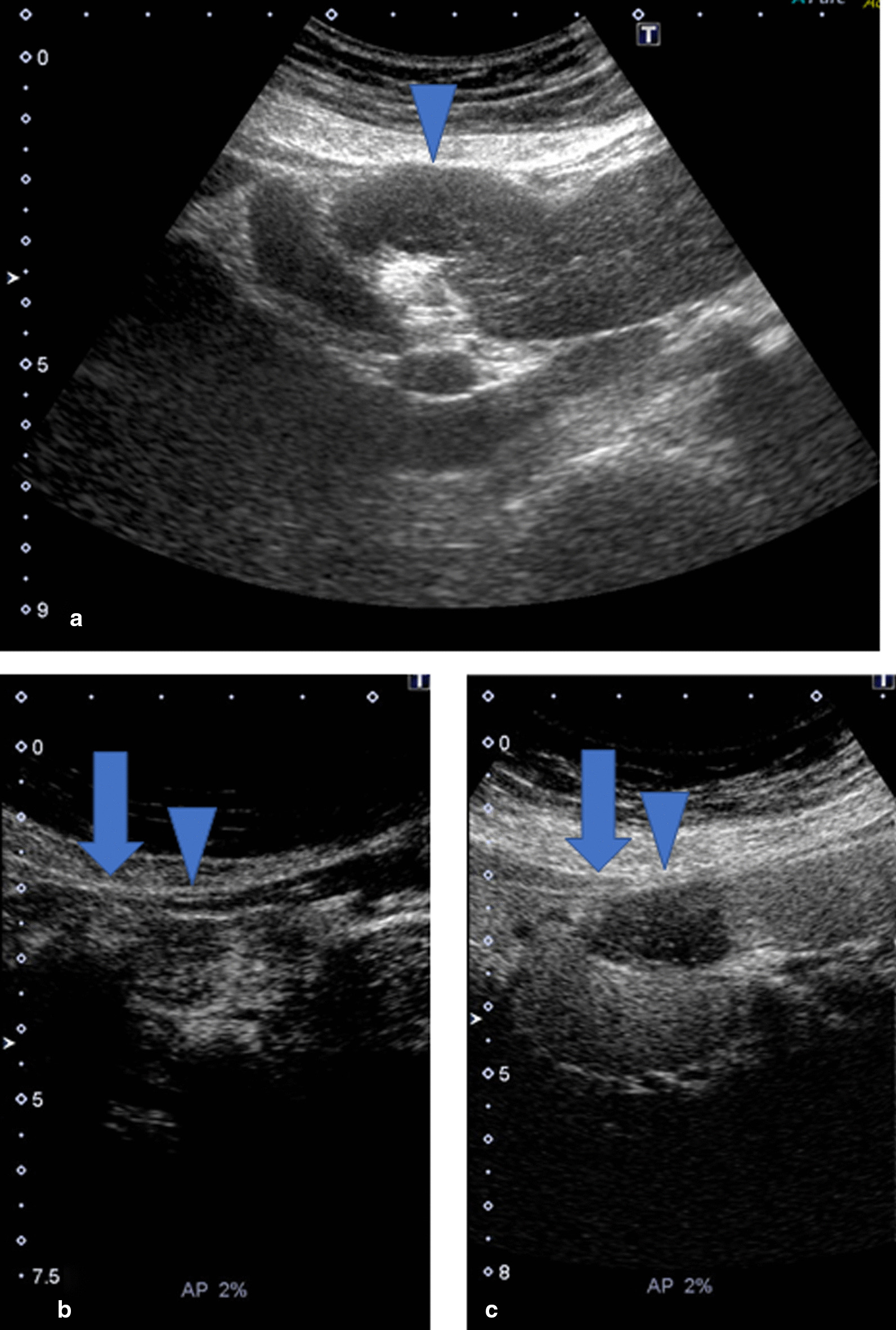
Ultrasound images of the tumor. **a** Ultrasound (US) examination of the protrusion area (arrowhead) in B-mode. **b** Contrast-enhanced US (CEUS), arterial phase. **c** CEUS, Kupffer phase. In CEUS, the tumor appears to have two components. The intrahepatic tumor (arrow, **b** and **c**) shows early enhancement and prolonged enhancement in the Kupffer phase. In the extrahepatic component (arrowhead, **b** and **c**), only the tumor margin is enhanced. The inside of the tumor shows spotty enhancement in the early phase

**Fig. 3:**
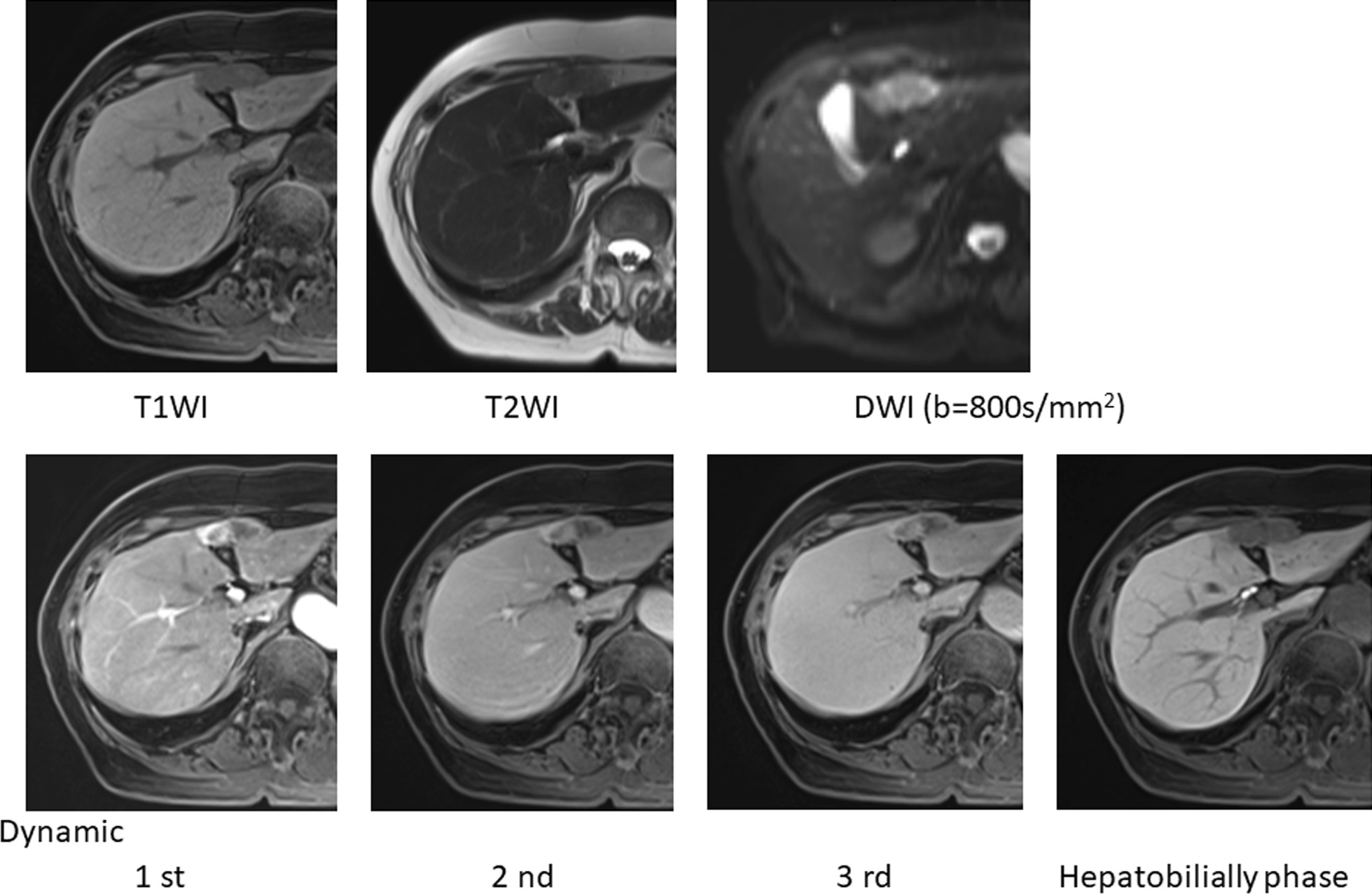
Gadolinium ethoxybenzyl diethylenetriamine pentaacetic acid magnetic resonance imaging images. The images indicate that the tumor has two components. *T1WI* T1-weighted images, *T2WI* T2-weighted images, *DWI* diffusion-weighted images

Ultrasound (US) also indicated that there were two components in one continuous tumor. US revealed that there was a hyperechoic component in the liver and that the protrusion area showed hypoechoicity (Fig. 2a). In contrast-enhanced US, the intrahepatic tumor showed vascularity in the early phase (Fig. 2b) and showed a defect in the Kupffer phase (Fig. 2c). In the extrahepatic protrusion, the surrounding area was strongly enhanced in a capsule shape with a spot-like inflow of contrast medium observed inside. Therefore, the extrahepatic region was considered to be a tumor component rather than a cystic structure such as an abscess or hematoma. Magnetic resonance imaging (MRI) with gadolinium ethoxybenzyl diethylenetriamine pentaacetic acid (Gd-EOB-DTPA) (Fig. 3) revealed that the tumor was seen 4 cm in a diameter, had a clear margin, and contained two components. The tumor showed low signal intensity on T1-weighted images and a slightly high signal intensity on T2-weighted images. T1-weighted dynamic contrast-enhanced MRI showed strong enhancement in the intrahepatic tumor and slight enhancement in the capsule-like outside of the extrahepatic tumor in the early phase. In the late phase, the intrahepatic tumor showed high-intensity at the peripheral rim and gradually showed low-intensity in other parts. Finally, each area clearly showed a defect in the hepatobiliary phase. In diffusion-weighted images, a slightly hyperintense signal in all areas of the tumor was observed.

In this case, the patient had no background history of hepatitis, obesity, alcohol, and diabetes. Additionally, there was no increase in the expression of tumor markers or inflammatory response, and the images were nonspecific, all of which made the diagnosis difficult. We initially suspected ICC in the liver tumor, but we could not explain the protrusion outside the liver. We realized that if the extrahepatic component was an abscess or hematoma, the blood flow inside the nodule was inconsistent. Furthermore, if the intrahepatic tumor was ICC, it was unlikely that it would rupture and form a hematoma outside the liver. We confirmed that there was no cholecystitis, and that the intestinal tract was not adhered, but we did not consider the mesenchymal lesions. Since imaging diagnosis did not lead to a definitive diagnosis of the tumor, we initially planned for diagnostic laparoscopic surgery. After laparoscopic observation, if an abscess was suspected, a partial excision was to be performed; if cancer was diagnosed after the excision, additional excision was to be performed in two stages if necessary. However, left hepatectomy was planned if ICC was suspected laparoscopically. After informing the patient of the likelihood of tumor growth and diagnostic purpose of the procedure, we obtained informed consent from the patient.

**Fig. 4:**
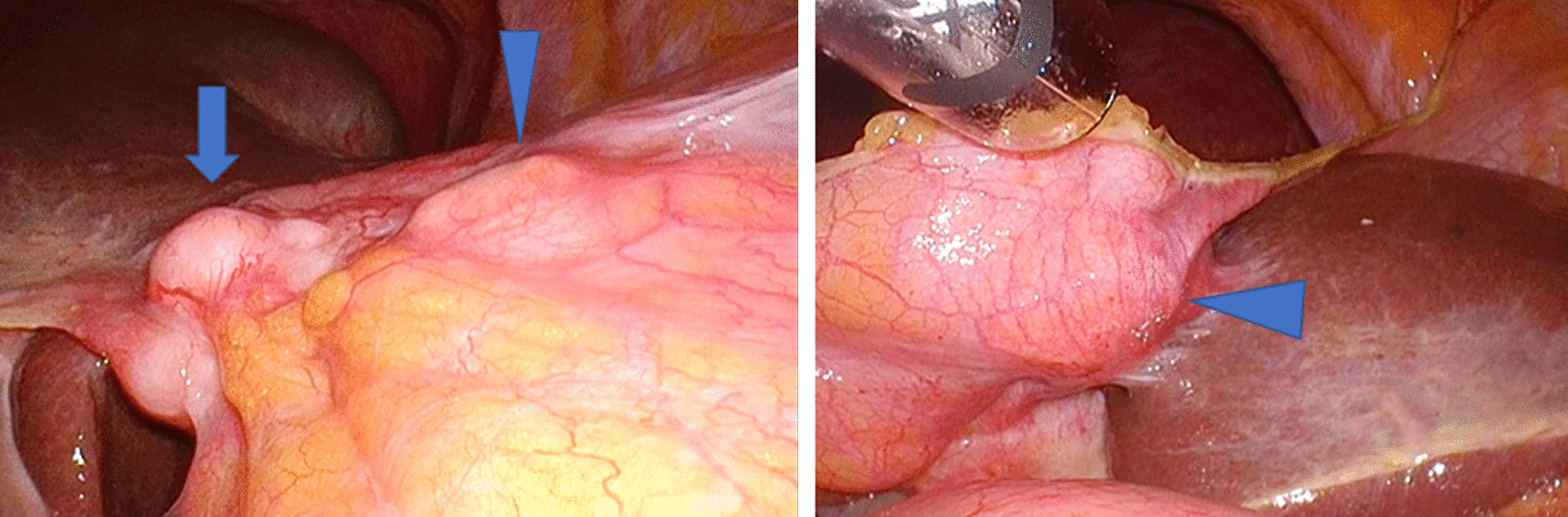
Observation view under laparoscope. A white nodule was visible on the surface of the falciform ligament (arrow), suggesting the presence of carcinoma. The mesentery was swollen and felt as if it contained a hard mass (arrowhead)

When observing the lesion laparoscopically (Fig. 4), a white nodule was visible on the surface of the falciform ligament, suggesting carcinoma. The mesentery was swollen and felt as if it contained a hard mass. Intraoperative US observation also confirmed the presence of a mass in the falciform ligament. By direct laparoscopic observation, we determined that the lesions identified as extrahepatic lesions by preoperative imaging were malignant tumors. ICC originates from the endothelial cells of the segmental or proximal branches of the bile duct. Unfortunately, ICC has a high incidence of locoregional recurrence even after surgery. Couinaud’s segments, sectors and hemilivers resection are recommended to carried out if the degree of liver fibrosis and future liver remnant volume are acceptable. In our patient, the total liver volume was estimated to be 830 mL, and the excision volume was estimated to be 31%. Since the residual liver volume was 570 mL (69%), it was judged that left hepatectomy was possible.

Left hepatectomy and cholecystectomy were performed laparoscopically. The patient experienced no postoperative complications and was discharged home 10 days after the operation.

**Fig. 5:**
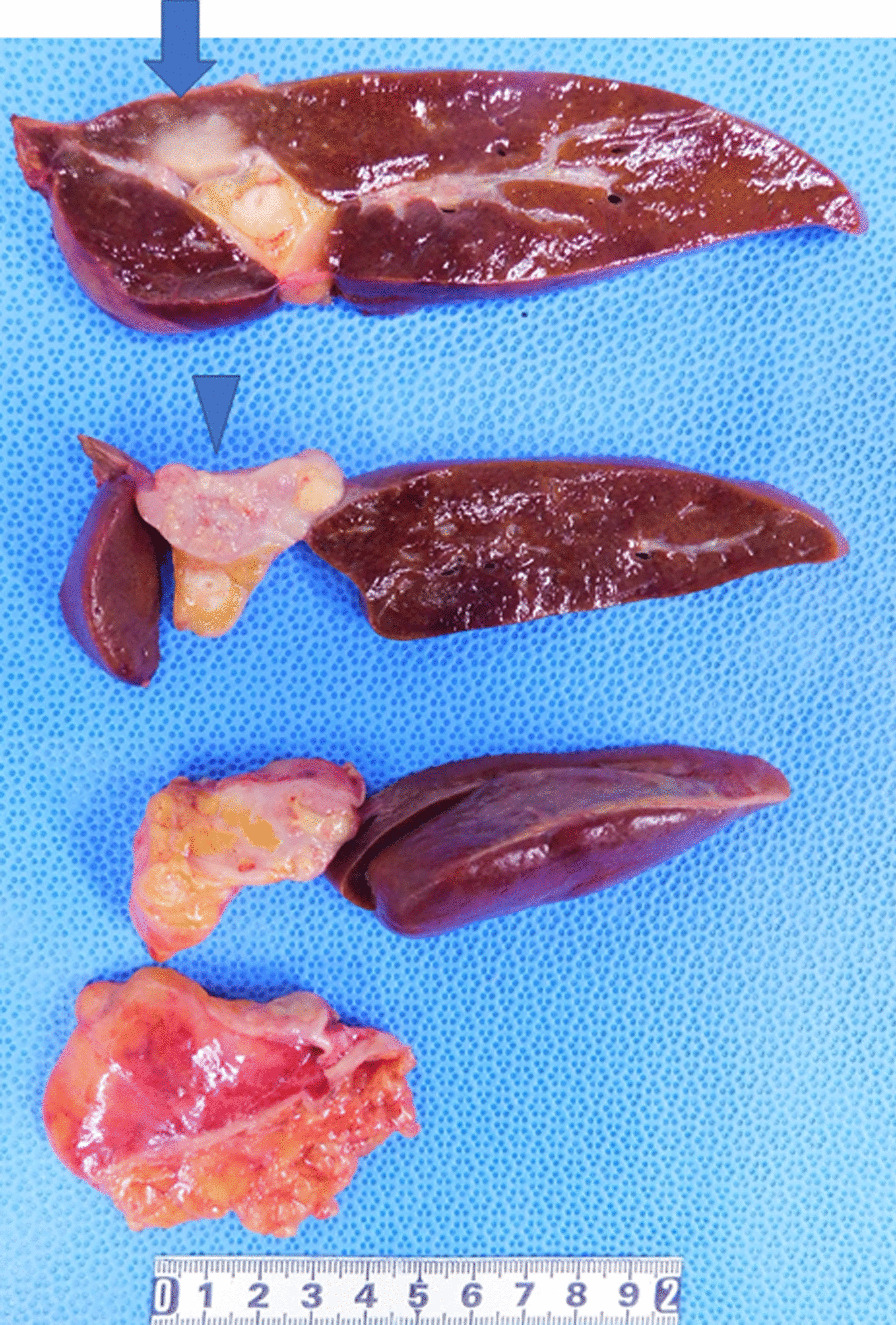
Tumor nodules. There was a 15 mm white nodule in the liver (arrow) and a 29 mm white nodule in the mesentery (arrow head). No continuity was found between the two tumors. Surgical margin was 15 mm

Macroscopically, there was a 15-mm white nodule in the liver and a 29-mm white nodule in the mesentery (Fig. 5). There was no continuity between the two nodules. Tumors in the liver were fibrous, hard, and non-capsular, whereas those in the mesentery were capsular and soft, with internal hemorrhage accompanying the capsule.

**Fig. 6:**
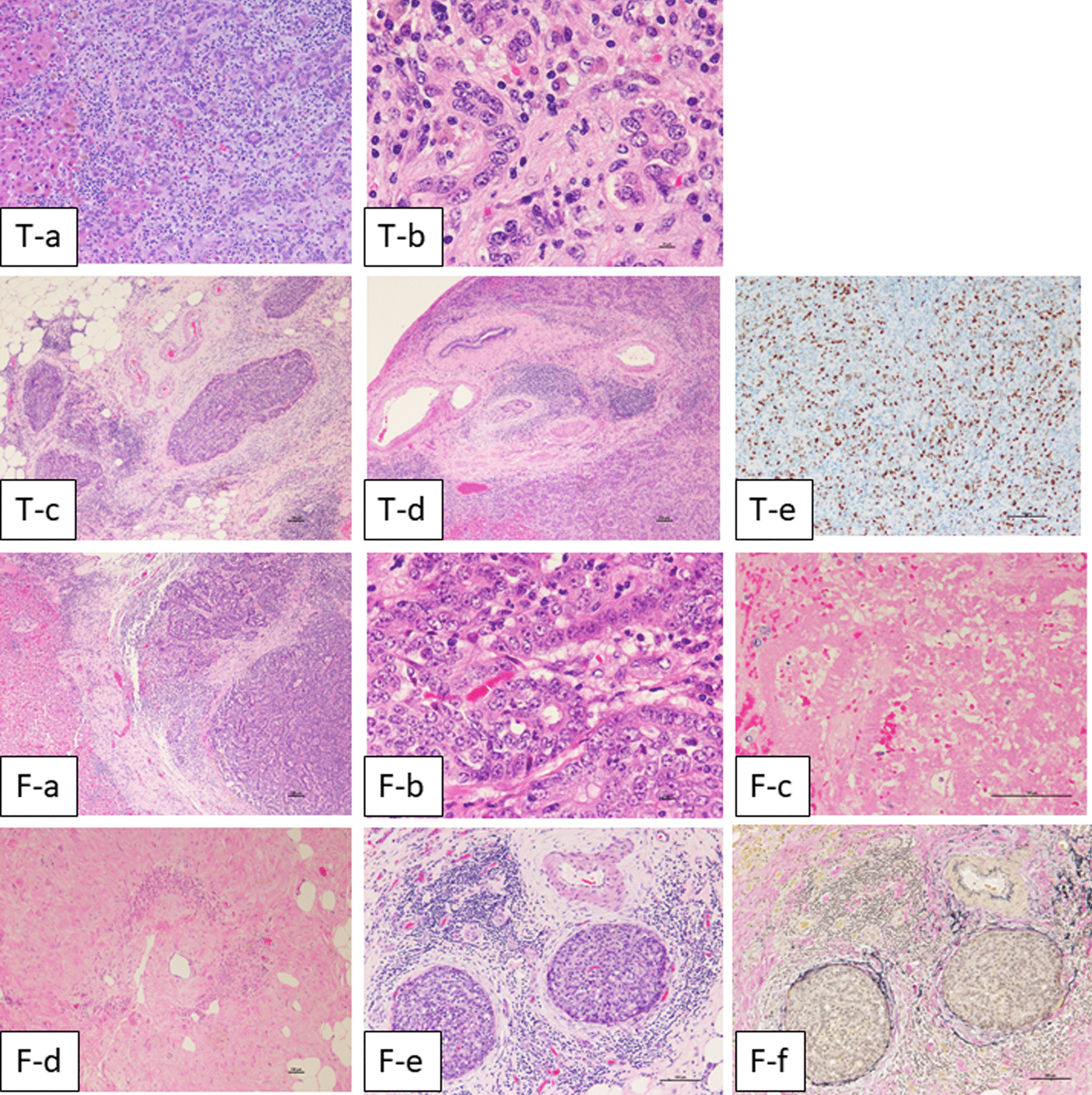
Microscopic findings. **T-a** Low-power field of hematoxylin eosin stain. **T-b** High power field of hematoxylin eosin stain. **T-c** Vascular invasion around the main tumor. **T-d** Neural invasion. **T-e** Ki-67 staining. **F-a** Low-power field of hematoxylin eosin stain in border area between the liver and mesentery. No direct infiltration was found. **F-b** High power field of hematoxylin eosin stain. **F-c** The center of the tumor showed extensive necrosis. **F-d** No tumor invasion was found in the round ligament. **F-e** Several tumor thrombi were found in the small veins of the falciform ligament. **F-f** Elastica van Gieson stain

Microscopically, the tumor showed glandular and papillary growth patterns with small amount of fibrosis and infiltration of the inflammatory cells (Fig. 6). The neoplastic cells were columnar and cuboidal and showed high nucleo-cytoplasmic ratio. The tumor was diagnosed as an ICC (Fig. 6T-a, b). Multiple vascular invasions were observed around the tumor (Fig. 6T-c) and neural invasion was also observed (Fig. 6T-d). The Ki-67 score was high, at 57 % (Fig. 6T-e). The structures of the extrahepatic tumor were histologically similar to that of the intrahepatic main tumor (Fig. 6F-a, b). The extrahepatic tumor had extensive necrosis in the center (Fig. 6F-c) and viable cells remained only in the peripheral area. There were no infiltrates in the round ligament of the liver (Fig. 6F-d), and several tumor thrombi were found in the small veins of the falciform ligament (Fig. 6F-e, f). Overall, the extrahepatic tumor was diagnosed as hematogenous metastasis of ICC. The preoperative CT scan was unclear because no angiography was performed; however, falciform ligament artery (FLA) seems to be branched from A4 on arterial phase of contrast-enhanced CT.

## Discussion and conclusion

The anatomy and variation of the falciform ligament is well defined, but the associated state of the falciform ligament remains unclear. The falciform ligament divides the medial and lateral segments of the liver, is located at the midline of the abdomen, and runs through the anterior wall of the abdomen and diaphragm [3]. It is well established that the arterial supply of the falciform ligament itself originates from a branch of the right hepatic artery (RHA), which is anastomosed to the superficial inferior epigastric artery [4]. That falciform ligament mainly contains the round ligament of the liver, which is a remnant of the umbilical vein from the fetal period, but also includes the hepatic FLA and falciform ligament vein (FLV).

The detection rate of FLA has been reported to be 2–42% by selective hepatic arteriography. The FLA arises as a terminal branch of the left (A2, A3, A3/4: 22–44%) or middle (A4: 56–77%) hepatic artery [5, 6]. It runs within the hepatic falciform ligament with the umbilical vein, provides partial blood supply around the umbilicus, and anastomoses with branches of the internal thoracic, inferior phrenic, and superior epigastric arteries [7, 8]. Although FLA is not routinely discussed in hepatectomy, it has been reported as a skin complication in transcatheter arterial chemoembolization for the treatment of hepatocellular carcinoma. Further, abscess formation and necrosis have been reported in falciform ligament [3, 9–11]. However, these are due to the umbilical arteries or veins, which are usually affected by the surrounding inflammatory disease such as pancreatitis or cholangitis. Alternatively, because the branch of the RHA nourishes the falciform ligament, cholecystitis may cause intramembrane abscess formation. [3, 9–11]. Despite various reports, it is still difficult to make a differential diagnosis of mass formation in the falciform ligament by imaging because there are still missing links in the etiology of tumor metastasis, invasion, and abscess formation, etc. [12]. Laparoscopic surgery including laparoscopic observation is very useful when a definite diagnosis cannot be made through imaging alone [3, 4, 11, 13].

In the present case, the preoperative images did not lead to an accurate diagnosis, and surgery was performed after confirmation by laparoscopic observation. The intramembranous tumor had the same histopathology as the main lesion and was consistent as a metastatic lesion. Additionally, many other micro-intravenous invasions were found in the falciform ligament. All of these were considered to be an intravenous invasion of the tumor into the drainage vein contained in the falciform ligament.

Considering the previous reports, tumors present in the left lobe, especially segment IV, may metastasize to the falciform ligament via intramembrane FLV. Even tumors located in the right lobe may metastasize to the falciform ligament because the feeding vessels of the membrane are branched from the RHA.

Recognizing this metastatic pathway is important because it can be helpful in preoperative diagnosis options; and it is also important for establishing surgical methods to control cancer.

In ICC, the exact role of post-resection systemic chemotherapy has not been established and it is not recommended in the guidelines due to insufficient evidence available for the use of adjuvant therapy. To date, surgical resection is the only potentially curative treatment for ICC [1, 2]. ICC is also well known to have a high recurrence rate. Prognostic factors for recurrence are vascular invasion, multiple tumors, and lymph node metastases. Consistent with these findings, although the main tumor was small in size, many tumor trombi were found in our patient. Six months have passed since hepatectomy in our patient, and no recurrence has been observed to date. However, from the pathological results, it is expected that hematogenous metastasis is more likely than intrahepatic metastasis or lymph node metastasis, so it is necessary to follow-up the patient for any signs of distant metastasis, such as in lung.

Our observations suggest that simultaneous excision of the falciform ligament and ligation alone may contribute to the suppression of metastasis. Moreover, histological examination of the membrane may help in clarifying the correlation between falciform ligament and intravenous distant metastasis.

## Data Availability

All data generated or analyzed during this study are included in this published article.

## Abbreviations

ICC: Intrahepatic cholangiocarcinoma
US: Ultrasound
CT: Computed tomography
CEUS: Contrast-enhanced ultrasound
Gd-EOB-DTPA: Gadolinium ethoxybenzyl diethylenetriamine pentaacetic acid
MRI: Magnetic resonance imaging
FLA: Falciform ligament artery
FLV: Falciform ligament vein
RHA: Right hepatic artery
